# Supplementary material for: Multi-carrier information hiding based on projection-driven vertex embedding in 3D models
Source: PLoS One. 2025 Sep 26;20(9):e0331588. doi: 10.1371/journal.pone.0331588 (PMC12469212; doi:10.1371/journal.pone.0331588)
Supplement: Attachment — Submitted filename: Response to Reviewers.docx [file pone.0331588.s002.docx]

**Reviewer #1:comments:**

**Comments 1:** The abstract should include some of the numerical results to relect the superiority of the proposed steganography algorithm.

**Response 1:** To enhance the robustness of single 3D model carriers in information hiding, this paper proposes a multi-carrier steganography algorithm based on vertex projection of 3D models. The algorithm improves the embedding capacity and attack resistance by fusing multiple 3D models into a geometrically invariant space using centroid coincidence and tangent plane projection. Secret information is embedded by adjusting the position of central vertices in the projection plane of their 1-ring neighborhoods. Experimental results demonstrate that the proposed method achieves strong robustness against translation, rotation, simplification, random noise, and shear attacks. Specifically, the proposed algorithm achieves a peak SNR of 47.87 dB on the xyzrgb_dragon model, significantly outperforming other algorithms, and maintains a lower BER under various attack intensities—for instance, less than 0.1 under 30% simplification and 0.001 random noise. These results confirm the superior invisibility and robustness of the proposed multi-carrier information hiding scheme.

**Comments 2:** The mentioned references in the introduction section are not very recent. The authors are encouraged to enrich their introduction section with the following list of recent and directly related works:

a.https://ieeexplore.ieee.ora/document/10643045

b.https://ieeexplore.ieee.org/document/10323523

c.httos://ieeexplore.ieee.org/document/10163067

d.https://link.springer.com/article/10.1007/s11042-020-09437-W

e.https://link.springercom/artide/10.1007/s00530-023-01253-0?fromPaywallRec=false

**Response 2:** Thank you for raising this reference question, which is really helpful for my paper. I have already cited three papers mentioned on websites B, C and D in the original article.

**Comments 3:** Figure 1 should be provided at a higher resolution. Currently, it is hard to read the information depicted on it.

**Response 3:** Thank you for your question. This is the best picture so far.

**Comments 4:** In the condusions section:a.Include some of the numerical results to reflect the superiority of the proposed algorithm.b.What are the limitations of this algorithm.

**Response 4:** In this paper, we propose a multi-carrier information hiding algorithm based on vertex projection of 3D models to address the limitations of embedding capacity and robustness in single-carrier steganographic schemes. By fusing multiple 3D models into a centroid-aligned and geometrically invariant fusion carrier, and embedding secret information through projection-based vertex manipulation, the algorithm significantly enhances both invisibility and resistance to geometric attacks.

Experimental results demonstrate the superiority of the proposed method. In terms of invisibility, the algorithm achieves a peak signal-to-noise ratio (SNR) of 47.87 dB on the xyzrgb_dragon model, which outperforms existing methods such as the SSC (46.58 dB), TSPD (45.32 dB), and ZWRA (44.79 dB) algorithms. Regarding robustness, the proposed method maintains a bit error rate (BER) lower than 0.1 under 30% vertex simplification, and remains resilient under random noise levels up to 0.001, with BER performance consistently superior to competing algorithms.

Nevertheless, the algorithm has certain limitations. Specifically, while it performs well under translation, rotation, simplification, and noise attacks, its robustness against large-scale shear attacks is relatively weak. This is mainly due to the absence of redundant embedding or multi-location replication strategies, which results in partial information loss when critical vertices are sheared away. Moreover, the algorithm assumes precise alignment during multi-carrier fusion and relies on geometric integrity, which may not hold under extreme deformations or lossy transmission channels.

Future work will focus on enhancing the algorithm’s resistance to destructive attacks such as large-scale shearing and affine distortions, potentially by introducing redundant encoding, adaptive region replication, or learning-based reconstruction to preserve information completeness and integrity under more adverse conditions.

**Reviewer #2:comments:**

Reviewer 2: About the manuscnipt "mult-carier information Hiding Algorithm Based on vertex proection of 3D Mode", the euthors present a multi-carierinformation hiding algorithm based on vertex projection of 3D models to improve the robusiness of sinale 3D model as a carrier for informationtiking. think this theme is more interesting, Overal, it sounds technicaly feasilble, and the structure of the manuscript is complete. Yet, here are some suggestionswhich should be taken into account to improve the current version.

**Comments 1：**Multrcarrier, but there doesn't seem to be much in the text

**Response 1:** (Introduction) at the end. In this work, the term multi-carrier refers to the use of multiple 3D models as concurrent carriers to overcome the embedding capacity and robustness limitations inherent in single-carrier schemes. Unlike traditional approaches that rely on a single model to embed all secret information, the proposed algorithm constructs a fusion carrier by geometrically aligning multiple 3D models through centroid coincidence and bounding sphere normalization. This multi-carrier strategy not only expands the available embedding region, thereby enabling higher-capacity information hiding, but also introduces spatial redundancy, which significantly enhances robustness against localized attacks. As a result, the embedded data is distributed across several carriers, making the system more resilient to partial model loss or deformation. Subsequent sections provide a detailed description of the multi-carrier fusion process and demonstrate its impact on the algorithm’s performance through experimental validation.

Additional sentence for the Conclusion:

The integration of multiple carriers plays a pivotal role in improving the embedding capacity and attack resilience of the system, serving as a fundamental enhancement over conventional single-model steganographic techniques.

**Comments 2：**From the references, most of them are relatively cld, so it is suggested to briefly discuss them with some new work

DOI :10.3390/math12243917;

DOI:10.1016/j.eswa.2024.123190;

DOI:10.1007/s11071-021-06206-8:

DOI:10.1007/900371-023-02812-2

**Response 2:** [23]Zhou, Y., Li, C., Li, W. et al. Image encryption algorithm with circle index table scrambling and partition diffusion. Nonlinear Dyn 103, 2043–2061 (2021). <https://doi.org/10.1007/s11071-021-06206-8>

[24]Feng, W., Zhang, J., Chen, Y., Qin, Z., Zhang, Y., Ahmad, M., & Woźniak, M. (2024). Exploiting robust quadratic polynomial hyperchaotic map and pixel fusion strategy for efficient image encryption. *Expert Systems with Applications*, *246*, 123190.

**Comments 3：**The curent experimental analysis is too simple, the research topic is informaion Hiding Algorithm, and a richer experimental comparison is recommendedacecrding to the analysis method in literature 15.

**Response 3:** To enable more comprehensive experimental comparisons, future work will further explore the following aspects of experimental comparison and analysis. Due to space constraints in this paper, these aspects will not be discussed here.

(1) Embedding Capacity Testing

Test the performance of different 3D models under various embedding rates.

Record the number of vertices and embedded bits for each model to calculate embedding capacity.

(2) Imperceptibility Testing

Use the Signal-to-Noise Ratio (SNR) to measure the distortion between the stego model (containing secret information) and the original model.

Visually compare the differences between embedded and original models.

(3) Robustness Testing

Translation Attack: Measure the Bit Error Rate (BER) after attacks with varying translation distances.

Rotation Attack: Measure the BER after attacks with different rotation angles.

Simplification Attack: Measure the BER under varying simplification rates.

Random Noise Attack: Measure the BER after adding random noise of different intensities.

Cropping Attack: Measure the BER under different cropping rates.

(4) Anti-Steganalysis Testing

Use existing steganalysis tools to analyze stego models and evaluate the algorithm's anti-steganalysis capability.

(5) Computational Complexity Testing

Record the algorithm's running time on 3D models of varying scales to evaluate time complexity.

Record the maximum memory consumption during execution to assess space complexity.

(6) Comparison with Other Algorithms

Select representative information hiding algorithms (e.g., those mentioned in Reference 15) for comparison under identical experimental conditions.

**Comments 4：**for the information hiding method, whether its securty can resist the attack of cryptanalysis can not be ignored, it is suggested to make a brief statement of thecryptanelysis wark.

DOI:10.1016/j.eswa.2024.123748

DOI:10.1016/i.eswa.2023.121514

**Response 4:**In addition to robustness metrics, preliminary observations suggest that the multi-carrier embedding strategy and the use of entropy-based encoding schemes (e.g., Huffman coding) offer a certain level of resilience against basic steganalysis and statistical cryptanalysis. Nonetheless, a comprehensive evaluation under advanced cryptanalytic models (e.g., chosen-message attacks, stego-only attacks) [25-26]is not yet conducted and will be considered in future research.

[25]Wen, H., Lin, Y., Yang, L., & Chen, R. (2024). Cryptanalysis of an image encryption scheme using variant Hill cipher and chaos. *Expert Systems with Applications*, *250*, 123748.

[26]Wen, H., & Lin, Y. (2024). Cryptanalysis of an image encryption algorithm using quantum chaotic map and DNA coding. *Expert Systems with Applications*, *237*, 121514.

**Comments 5：**The curent article draft stllcontains a significant number of typographical and grammatical errors. Additinally, it is recommended to study the writing andexpression of higher-level literature, and to carefully check and correct these issues.

**Response 5:**Thank you for your valuable feedback and for highlighting the importance of rigorous language and formatting standards. We sincerely apologize for the oversights in the initial submission and have taken comprehensive steps to address these issues. Should any residual issues be identified, we remain fully committed to addressing them promptly.

**Reviewer #3:comments:**

Reviewer #3:Review commendsThank the authors for the submision of the manuscript entitled "Mult-carner Information Hiding Algorthm Based on Vertex Projection of 3D Model" The paperneeds more work to achleve a publlshable level due to the following reasons.

**Comments 1：**Motivation and objective of the paper is missing.

**Response 1:**Paragraphs 4 and 5 of Introduction

Although a variety of 3D model-based information hiding algorithms have been developed, most existing approaches suffer from limited embedding capacity and poor robustness against geometric attacks, especially when using single-carrier models. These limitations restrict their applicability in real-world scenarios where data integrity and confidentiality under complex transmission environments are critical.

To address these challenges, this paper is motivated by the need to enhance both the robustness and embedding capacity of 3D steganographic systems. The objective of this work is to develop a multi-carrier information hiding algorithm that leverages the geometrical fusion of multiple 3D models and vertex neighborhood projection, allowing for reliable and covert communication under various attack scenarios, including translation, rotation, noise, simplification, and shear.

**Comments 2:**What is the contribution of this paper?

**Response 2:** The last paragraph of Introduction

The main contributions of this paper are summarized as follows:

A multi-carrier information hiding framework is proposed, which fuses multiple 3D models through centroid alignment and geometric normalization. This fusion overcomes the capacity limitations and improves the redundancy of traditional single-carrier schemes.

A vertex projection embedding method is designed, in which the secret information is hidden by adjusting the central vertex position in the 1-ring neighborhood projection plane. This approach enhances the algorithm's resistance to translation, rotation, and noise attacks.

A candidate vertex selection strategy based on vertex importance and 1-ring neighborhood area is introduced to balance invisibility and robustness, ensuring that embedding occurs in stable regions of the mesh.

Extensive experiments on benchmark 3D models demonstrate that the proposed algorithm achieves superior robustness and invisibility, with SNR values up to 47.87 dB and low BER under various attack scenarios, outperforming several state-of-the-art methods.

**Comments 3:** Authors have not included some recent existing paper from year (2023 and 2025) in introduction Part. So, Authors must cite some recent work related in the open.

i. A cellular automata based secured reversible data hiding scheme for dual lmages using bit-reversal permutation technlgwe, k Datta, 8 jana, Mp chakraborty.Computer standards & Interfaces 92.103919.2025.
ii. Zhou, G. liu, w, 2hu, 9. Lu, Y, & iu, Y (2022), ECA-MobileNetV3(arge)+SegNet Modelfor Binary Sugarcane classification of Remotely Sensed Image, IETransactlons on Geosclence and Remote Sensing, 60. dol: 10.1109/TGRS.2022.3215802i.
iii. shl, H., Da0, s. ...8 Cal, 1.(2025). LLMormer: Large Language Model for Open-vocabulary semantic segmentation. interational joumal of Computer Vislon,133(2)，742-759.doi:10.1007/s11263-024-02171-y
iv. Reversible data hiding strategy exploliting circular distance interpolation utllzing optimal pixel adjustment with error substitution, M Jana, 8 jana, s jardarMultimedia Tools and Applications 83(16),48949-48986,22024.
v. Context pixel-based reversible data hiding scheme using pixel value ordering, s Meikap, B Jana, Tc lu, The Visual computer 40 (5), 3529-3552, 102024
vi. Dual image based secured reversible data hiding scheme exploiting huffman compression tree combining bit-reversal permutation technique
vii. Hlgh-Payload kDH Technlque for Secure Data Transmission Through lmproved Context pixel-Based Pv0 Explolting Center-folding Strategy
viii. A Weighted Matrix-Based Reversible Data Hiding scheme with Dual-image by Exploiting BWT Encoding Technique
ix. .A Dual-Ima9e: Based Secured Reversible Data Hiding Scheme Exploiting Weighted Matrix and Cellular Automata
x. AMBTc-Based High capacity Data Hiding Scheme Exploitina PVD and BRP
xi. Reference pixel-based reverslble data hlding scheme using multl-level pixel value ordering
xii. Robust data hidinassed image exploiting btc with hamming code
xiii. Dual-image reversibleencoding the numeral svstem of concealed inforrationnot cleary addressed the issues of the existing state of art techniques.

**Response 3:**The suggestions you put forward are very good. Based on the suggestions of other judges, I will appropriately add the participating literature to support my thesis views.

**Comments 4:** In Introduction Part, authors have not clearly addressed the issues of the existing state of art techniques.

**Response 4:**I have rephrased the introduction section:

(1) Limited Embedding Capacity

Most existing 3D model-based information hiding algorithms utilize a single 3D model as the carrier. The limited number of vertices and meshes available for embedding secret data results in insufficient embedding capacity.

(2) Insufficient Robustness

Single-model information hiding algorithms exhibit poor robustness against attacks such as translation, rotation, random noise, and simplification. This vulnerability makes the hidden information prone to loss or leakage.

(3) Security Concerns

Due to the susceptibility of 3D models to various geometric attacks during transmission, existing information hiding technologies struggle to ensure the security and integrity of secret information transmission.

**Comments 5:** Authors have not cleary highlght the technical contribution of thelr work, How your work is different from other published work in the similar direction? justify it

**Response 5:**Technical Contributions and Advantages

The technical contributions of this study are highlighted in three key innovations. First, the proposed multi-carrier fusion technique addresses the limitations of existing single-model information hiding methods, which suffer from restricted embedding capacity and vulnerability to robustness issues. By integrating multiple 3D models and expanding the embedding region through vertex projection, this approach significantly enhances both embedding capacity and robustness. Second, the geometric invariant space mapping mechanism improves resistance to geometric attacks (e.g., translation, rotation) by mapping fused models into an invariant space, ensuring stability under transformations—a critical advancement over traditional methods.

Third, the vertex selection and embedding strategy introduces a novel screening process based on vertex importance and 1-ring neighborhood area to identify robust candidate points. Unlike conventional methods that directly modify vertex positions in 3D space, this study projects candidate vertices onto tangent planes and embeds secret data by adjusting projected coordinates. This minimizes visual distortion while improving imperceptibility. Additionally, the algorithm’s robustness is rigorously validated through diverse attack experiments (e.g., noise, simplification, cropping), demonstrating superior performance compared to existing approaches.

In summary, this study advances 3D model-based information hiding through multi-carrier fusion, geometric invariant mapping, and adaptive vertex selection/embedding techniques. These innovations collectively address critical challenges in embedding capacity, robustness, and security, offering a marked improvement over state-of-the-art methods. Experimental results confirm the algorithm’s resilience to attacks and its practical superiority in real-world scenarios.

**Comments 6:** How much intormation we canhide inside the multimedia obiect, alona with acceotable visual quality?

**Response 6:**The proposed information hiding algorithm in this study embeds secret data into 3D models while maintaining acceptable visual quality through the following key techniques: multi-carrier fusion, geometric invariant space mapping, vertex importance and 1-ring neighborhood area screening, and vertex projection-based embedding. The workflow comprises the following steps:

(1) Multi-Carrier Fusion Technique

Fuse multiple 3D models into a unified multi-carrier fusion state to expand the embedding region and enhance embedding capacity.

Distributing secret information across multiple models reduces modifications to individual models, minimizing visual distortion.

(2) Geometric Invariant Space Mapping

Map the fused model into a geometric invariant space to resist geometric attacks (e.g., translation, rotation) while preserving visual quality.

(3) Vertex Importance and 1-Ring Neighborhood Area Screening

Select candidate vertices for embedding based on their importance and 1-ring neighborhood area.

Prioritize vertices with high importance and moderate neighborhood areas to avoid regions prone to noticeable visual impact.

(4) Vertex Projection and Secret Information Embedding

Project the 1-ring neighborhood of candidate vertices onto tangent planes.

Embed secret information by adjusting the position of the central vertex within the projection plane, minimizing alterations to the 3D model’s visual appearance.

(5) Experimental Validation

Validate robustness against attacks including translation, rotation, random noise, and simplification.

Results demonstrate that the algorithm maintains strong robustness while preserving high visual quality.

By integrating these methods, the proposed algorithm effectively conceals secret information in 3D models and ensures acceptable visual fidelity throughout the embedding process.

**Comments 7:** I suggest the authors to take carerecuncancy, From the paper, I can see that some of the literature is discussed several times.

**Response 7:**In this paper, several references are cited multiple times. The details of these repeated citations are as follows:

(1) Reference [17]

First mention: In Section 2.4 ("Secret Information Embedding Rules"), stated: "The tangent plane of a vertex’s 1-ring neighborhood is unique [17]."

Second mention: In Section 3.1 ("Imperceptibility Experiment"), stated: "Comparative experiments were conducted with algorithms from References [17, 18, 19]."

(2) Reference [18]

First mention: In Section 3.1 ("Imperceptibility Experiment"), stated: "Comparative experiments were conducted with algorithms from References [17, 18, 19]."

Second mention: In Section 3.2 ("Robustness Experiment"), stated: *"The same secret information was embedded, resulting in 16 stego carriers. Since the complete extraction of secret information after model encryption in complex environments is critical, the robustness of different methods was evaluated using the Bit Error Rate (BER), defined as the ratio of erroneous bits to the total number of secret bits, with a range of [0, 1]. A BER closer to 0 indicates fewer errors and stronger robustness, as calculated by Equation (7)."

(3) Reference [19]

First mention: In Section 3.1 ("Imperceptibility Experiment"), stated: "Comparative experiments were conducted with algorithms from References [17, 18, 19]."

Second mention: In Section 3.2 ("Robustness Experiment"), stated: "The same secret information was embedded, resulting in 16 stego carriers. Since the complete extraction of secret information after model encryption in complex environments is critical, the robustness of different methods was evaluated using the Bit Error Rate (BER), defined as the ratio of erroneous bits to the total number of secret bits, with a range of [0, 1]. A BER closer to 0 indicates fewer errors and stronger robustness, as calculated by Equation (7)."

**Comments 8:** Can you elaborate on the specific challenges faced by existing RDH methods, especially in.

**Response 8:**The existing reversible data hiding (RDH) methods discussed in this paper face the following specific challenges:

(1) Limited Embedding Capacity: The finite number of vertices and meshes in a single 3D model restricts the amount of secret information that can be embedded.

(2) Insufficient Robustness: Current RDH methods exhibit poor robustness against attacks such as translation, rotation, random noise, and simplification, which often lead to information loss or leakage.

(3) Degraded Visual Quality: To maintain robustness, some methods may introduce noticeable distortion to 3D models, compromising visual quality.

(4) High Computational Complexity: Complex processes like coordinate transformations and vertex importance calculations increase time and space complexity, making these methods impractical for large-scale applications.

(5) Weak Anti-Analysis Capability: Existing approaches perform poorly in resisting steganalysis; steganalysis tools can detect model modifications, thereby exposing hidden data.

(6) Inadequate Robustness Against Specific Attacks: While demonstrating reasonable resistance to translation, rotation, and random noise attacks, these methods show insufficient robustness against specific attacks (e.g., cropping attacks), resulting in information loss.

These challenges highlight significant room for improvement in existing RDH methods regarding embedding capacity, robustness, visual quality preservation, computational efficiency, and resistance to advanced attacks.

**Comments 9:** How to measure the effectiveness of your proposed method?

**Response 9:**The effectiveness of the information hiding algorithm proposed in this paper can be evaluated through the following aspects:

(1) Imperceptibility

Signal-to-Noise Ratio (SNR): The imperceptibility of the algorithm is measured by calculating the SNR between the stego model (containing secret information) and the original model. A higher SNR indicates better imperceptibility and less visual distortion.

(2) Robustness

Bit Error Rate (BER): After subjecting the stego model to various attacks (e.g., translation, rotation, simplification, random noise, and cropping), robustness is evaluated by calculating the ratio of erroneous bits to the total bits in the extracted secret information. A BER closer to 0 signifies stronger robustness.

(3) Embedding Capacity

By leveraging the multi-carrier fusion technique, the algorithm expands the embedding region and dynamically adjusts the number of carriers based on the size of the secret information, making it suitable for high-capacity application scenarios.

(4) Anti-Analysis Capability

The algorithm selects vertices with moderate 1-ring neighborhood areas as carriers and embeds secret information in projected tangent planes. This minimally alters the 3D spatial positions of vertices, thereby enhancing resistance to steganalysis.

(5) Computational Complexity

Time Complexity: The algorithm exhibits a time complexity of *O* (*N*), where *N* is the total number of vertices in the model.

Space Complexity: The space complexity depends on the model size, requiring sufficient memory allocation to store all vertex data.

Through a comprehensive evaluation of these metrics, the effectiveness of the proposed information hiding algorithm is rigorously validated.

**Reviewer #4:comments:**

Reviewer #4: Multi-carrier Information Hiding Algorithm Based on Vertex Projection of 3D. The manuscript explained advancements of 3D model as a carrier for information hiding, a mult-carier information hiding algorithm based on vertex projection.However there are stll some issues that need to be considered for further enhance the quality of the manuscrit, The reviewer addressed the comments:

**Comments 1:** The manuscript title and Reference [6] title are same. So need to change the title and check the plagiarism before sending to the reviewer.

**Response 1:**Multi-Carrier Information Hiding Based on Projection-Driven Vertex Embedding in 3D Models

**Comments 2:**The authors should recheck the references related to the AScAD database. i believe that references [7] and [8] are the same.

**Response 2:**Your question is very good. I checked the papers published by the same author in different years.

**Comments 3:**The authors need to revise the introduction in section 1. The introduction are wel descnbed by the apprach of existing Model. Try to add a paragraph related to reversible data hiding schemes

"A Review of Reversible Data Hiding Technique Based on Steganography", procedings ofthe ARpN journal of Engineering and Applied science, vol.13, N.3, Feb.2018,pp.1105-1114.

**Response 3:**Reversible data hiding schemes have also been extensively studied; for a comprehensive review, see [27].

[27]Raju, K. U., & Prabha, N. A. (2018). A review of reversible data hiding technique based on steganography. Proc ARPN J Eng Appl Sci, 13(3), 1105-1114.

Introduction should be included as, why the 3D projection model is applicable in the steganography model.

**Response:**The 3D vertex projection technique provides a stable geometric domain for information embedding, enabling high imperceptibility and robustness, which makes it well suited for steganographic applications.

**Comments 4:**literature survey should be included recent year research artidles related to 3D model in information hiding techniques. indude the folowing artice in reterence.

"Data hiding steganography model based on hyper chaos 2D compressive sensing inhabited with manchester encoder/decoder using circular queue exploiting modlfication direction" journal of Intelligent & Fuzzy Systems, Vo.44,2023,pp:10357-10367.

**Response 4:** [28]Upendra Raju, K., & Amutha Prabha, N. (2023). Data hiding steganography model based on hyper chaos 2D compressive sensing inhabited with manchester encoder/decoder using circular queue exploiting modification direction. *Journal of Intelligent & Fuzzy Systems*, *44*(6), 10357-10367.

**Comments 5:** lWhat are the attacks considered in the proposed techniques?

**Response 5:** The proposed technique primarily considers geometric attacks such as translation and rotation, as well as topological attacks like mesh simplification, which are common and challenging scenarios in 3D steganography.

**Comments 6:**Several points make this work interesting, but need to explain how the proposed method works in the system.

**Response 6:**Explanation of How the Proposed Method Works in the System

The proposed multi-carrier information hiding algorithm operates through a systematic workflow designed to enhance robustness, embedding capacity, and invisibility. Here is a step-by-step explanation of the method:

(1) Multi-Carrier Fusion

Multiple 3D models are fused into a unified geometric invariant space via centroid alignment and bounding sphere normalization. This fusion expands the embedding region and introduces spatial redundancy, distributing secret information across multiple carriers to reduce per-model distortion.

(2) Geometric Invariant Preprocessing

The fused model undergoes coordinate system redefinition, rotation/translation alignment, and uniform scaling to ensure invariance against geometric attacks (e.g., translation, rotation). This step guarantees consistent embedding and extraction regardless of geometric transformations.

(3) Vertex Selection and Simplification

Vertices are prioritized based on their importance (distance to the average plane of their 1-ring neighborhoods) and 1-ring neighborhood area. Vertices with moderate neighborhood areas are selected as embedding candidates to balance robustness against simplification and subdivision attacks.

(4) Projection-Driven Embedding

The 1-ring neighborhood of each candidate vertex is projected onto a tangent plane. Secret information is embedded by adjusting the central vertex’s position in the 2D projection, minimizing 3D spatial distortion. This ensures high imperceptibility while embedding data.

(5) Robust Extraction

During extraction, the fused model is reconstructed, vertices are re-screened, and secret bits are retrieved from the projection planes using geometric invariants (e.g., relative positions in the tangent plane).

The system’s novelty lies in its multi-carrier fusion (addressing capacity limitations) and projection-based embedding (enhancing geometric robustness). By distributing data across multiple models and leveraging invariant geometric features, the algorithm achieves resilience to attacks while maintaining visual quality.

**Comments 7:**The fiqures 9, 10, 11, 12 and 13 mentioned that TSPD,ZWRA and ssc algorithms with diferent colors, Mentioned the reference number corespond to theexisting algorithms while compare to the proposed algorithms.

**Response 7:**Consistency in Color Coding for Comparative Algorithms

Thank you for the valuable feedback. We acknowledge the importance of clarity in visual representations. In Figures 9–13, the TSPD [14], ZWRA [13], and SSC [15] algorithms were originally differentiated using distinct colors (e.g., blue, red, green) to highlight performance differences. However, to improve readability and align with your suggestion, we have revised the figures as follows:

Consistent Color Assignment:

TSPD (Triangular Strips Progressive Grid) [14]: Unified to blue.

ZWRA (Zero Watermark Rotation Attack) [13]: Unified to red.

SSC (Spatial Subdivision and Coding) [15]: Unified to green.

Proposed Method: Highlighted in purple for clear distinction.

Legends and References:

A standardized legend now explicitly maps each algorithm to its assigned color and corresponding reference number (e.g., “TSPD [14] – Blue”).

Footnotes in the figure captions reinforce the mapping (e.g., “Comparative algorithms: TSPD [14] (blue), ZWRA [13] (red), SSC [15] (green)”).

This revision ensures that readers can effortlessly correlate the algorithms with their cited references while maintaining visual coherence across all figures.

**Comments 8:** The simulated reult is otained eficienty but there is a lackin the justification in al simulated fioures and tables, mainly tables (2) and fqures 9, 10, 11, 12 and13.

**Response 8:** After Table 2 (SNR) ：

As shown in Table 2, the proposed algorithm achieves the highest SNR values across all tested models, indicating minimal distortion and strong invisibility compared to the baseline methods.

Figure 9–10（translation & rotation attack）：

The BER remains consistently low under both translation and rotation, validating the geometric invariance of the projection-based embedding scheme.

Figure 11（simplification attack）：

Even under 30% vertex simplification, the BER of the proposed method remains significantly lower than other algorithms, demonstrating strong robustness.

Figure 12–13（random noise attack）：

The BER increases more slowly under noise attacks in the proposed algorithm, suggesting its effectiveness in resisting vertex perturbation.

**Comments 9:** The provided simulative reults are completely related to this work eficiently,but to justify the contribution of the work and how much level of the proposed workwas improved compared to the existing methods must be incuded in conclusion.

**Response 9:** Add in the summary

Compared with existing methods such as SSC, TSPD, and ZWRA, the proposed algorithm improves the peak SNR by up to 1.3 dB, and reduces the BER under simplification and noise attacks by over 30%, demonstrating a significant enhancement in both invisibility and robustness.
